# UHPLC-Q-TOF/MS unveils metabolic diversity in *Paris* L. and highlights medicinal potential of five newly identified species

**DOI:** 10.3389/fphar.2025.1605264

**Published:** 2025-07-23

**Authors:** Kangmeng Sun, Yingying Shao, Rong Li, Zhi Wang, Jinghan Wang, Xinyu Luan, Changqiao Wu, Liya Wang, Chunnian He, Qi Tang

**Affiliations:** ^1^ State Key Laboratory for Quality Ensurance and Sustainable Use of Dao-Di Herbs, Institute of Medicinal Plant Development, Chinese Academy of Medical Sciences and Peking Union Medical College, Beijing, China; ^2^ Yuelushan Lab, College of Horiculture, Hunan Agriculture University, Changsha, China; ^3^ College of Pharmacy, Hunan University of Chinese Medicine, Changsha, China; ^4^ Resource and Environment College, Central South University of Forestry and Technology, Changsha, China

**Keywords:** *Paris* L. (Melanthiaceae), metabolomics, metabolic profiling, steroidal saponins, newly identified species, medicinal potential

## Abstract

*Paris* species are widely distributed across China, with their rhizomes traditionally used in Chinese medicine. *P*. *polyphylla* var. *chinensis* (PPC) and *P*. *polyphylla* var. *yunnanensis* (PPY) are listed in the official pharmacopeia and have been extensively studied, however, the metabolic diversity of other species within the genus remains largely unexplored. To address this gap, a comprehensive UHPLC-Q-TOF/MS-based metabolomic analysis was conducted on rhizomes from 26 *Paris* species and varieties, five of which have been published in recent years. Multivariate statistical analyses, including PCA and HCA, revealed three distinct metabolic groups. Group 1, dominated by pennogenin saponins, exhibited the highest overall steroidal saponin content. Group 2 displayed lower total saponin levels and marked metabolic heterogeneity, whereas Group 3 was characterized by a predominance of diosgenin saponins. Comparative analysis of the five newly identified species and the two pharmacopoeial species identified 24 common metabolites and 43 differentially accumulated metabolites. Notably, *P. xuefengshanensis* and *P. qiliangiana* contained relatively high levels of Polyphyllin I, II, and VII, with *P. xuefengshanensis* surpassing PPY and PPC. Additionally, *P. tengchongensis* exhibited notably high relative levels of Gracillin and Protogracillin. These findings highlight the presence of unique metabolites and relatively high concentrations of bioactive steroidal saponins in the newly identified species, suggesting their potential medicinal value, particularly for antitumor and hemostatic applications. This study provides new insights into the metabolic diversity of *Paris* species and supports the exploration of these under-researched resources for pharmacological use.

## 1 Introduction

The genus *Paris* L. (Liliales: Melanthiaceae) comprises *ca.* 26 species of perennial herbaceous plants widely distributed across East Asia and Europe, with 22 species found in China (Ji, 2021). Distinguished by a solitary stem, a whorl of typically seven leaves, and a single terminal flower, *Paris* plants are commonly known as “Seven-Leaf One-Flower.” The medicinal use of *Paris* species was first documented in Shennong’s Classic of Materia Medica during the Eastern Han dynasty and has been traditionally used among ethnic minorities to treat gastric ulcers, wounds, hemorrhages, and snakebites (Ding et al., 2021; Addi et al., 2024). In China, the rhizomes of *P*. *polyphylla* var. *chinensis* (PPC) and *P*. *polyphylla* var. *yunnanensis* (PPY) are officially recorded in the Chinese Pharmacopoeia as the sources of Rhizoma Paridis (China, 2020), which has been used in over 106 classic formulations for treating a wide variety of conditions. For example, *Gongxuening* capsules are used to treat uterine bleeding and chronic pelvic inflammatory disease, and *YunnanBaiyao* aerosol/capsules are employed for healing injuries and blood stasis swelling pain.

To date, more than 430 secondary metabolites have been isolated and identified from *Paris* plants, including steroids, terpenoids, flavonoids, and other compounds. Steroidal saponins are the primary bioactive constituents, accounting for over 80% of the identified compounds. Steroidal saponins can be further classified into spirostanol, isospirostanol, furostanol, and pseudospirostanol types based on the configuration of C-25 spirosteranes and the cyclic state of the F ring (Ye et al., 2024). Among them, pennogenin and diosgenin saponins, both subclasses of isospirostanol saponins, are key contributors to the pharmacological activity of Rhizoma Paridis, demonstrating anti-tumor, analgesic, anti-inflammatory, hemostatic, and antibacterial effects (Chen et al., 2021). Notably, Polyphyllin I, II, and VII are recognized as quality control markers for Rhizoma Paridis in the Chinese Pharmacopoeia (2020 edition), with a required combined content of no less than 0.60%.

Over the past decade, modern analytical techniques, particularly ultra-performance liquid chromatography coupled with quadrupole time-of-flight mass spectrometry (UPLC-Q-TOF/MS), have been widely applied to investigate secondary metabolites in *Paris* species. Studies indicated that the metabolite accumulation in five/nine *Paris* species, as well as three regions of PPC and PPY, was influenced by both species variation and geographical factors (Wu et al., 2018; Wang Y. Z. et al., 2017). Moreover, chemotaxonomic relationships based on metabolite profiles partially align with morphological taxonomy (Wang Y. Z. et al., 2017). For example, *P. mairei* shares a similar chemical profile with PPY, which could be treated as a proposed substitute for PPY (Yang et al., 2023). Further research has found that some species, including *P. delavayi*, *P. forrestii*, and *P. fargesii*, not only contain high levels of steroidal saponins but also exhibit promising pharmacological effects (Liu et al., 2012; Li, 2021; Sun et al., 2024). These findings suggest that, beyond PPC and PPY, other *Paris* species may also possess significant medicinal potential. However, current research remains largely focused on a few species such as PPC and PPY, leaving the metabolic profiles and bioactive properties of other species underexplored.

The pharmacophylogeny theory suggests that medicinal plants exhibit a correlation between phylogeny, chemical composition, and therapeutic effects, which has been widely applied to expand medicinal resources, enhance quality control, and predict bioactive constituents (Gong et al., 2022). It emphasizes that closely related species or genera tend to produce similar bioactive compounds, leading comparable medicinal properties. This approach is particularly useful for discovering alternative resources for rare and endangered medicinal plants. To date, the phylogenetic relationships of more than 24 phytogroups have been studied, including *Salvia*, *Scutellaria,* and *Dichocarpum* (Xiao et al., 2021). In the fourth national census of Chinese medicine resources (2011–2024), over 10 new *Paris* species and varieties were discovered, yet their metabolic profiles and medicinal potential remain largely unexplored. Therefore, based on phylogenetic relationships, a systematic metabolomics study could provide valuable insights into the metabolic diversity within the *Paris* genus and facilitate the discovery of novel medicinal resources.

In this study, 26 species and varieties of *Paris* were selected based on phylogenetic relationships, geographic distributions, and medicinal relevance, with the aim of maximize taxonomic coverage and capturing representative patterns of metabolic diversity across the genus. To systematically explore this diversity, the chromatographic retention behavior of steroidal saponin standards was first analyzed using UHPLC-Q-TOF/MS. Principal component analysis (PCA), hierarchical clustering analysis (HCA), and orthogonal partial least squares discriminant analysis (OPLS-DA) were then employed to characterize the metabolic profiles of 26 *Paris* species and varieties, enabling the identification of metabolite similarities and differences, and revealing intrinsic metabolic patterns. Additionally, common and differentially accumulated metabolites were compared between five newly identified species (*P. nitida, P. qiliangiana, P. xuefengshanensis, P. yanchi*, and *P. tengchongensis*) and two pharmacopoeial varieties (PPC and PPY), providing a scientific basis for evaluating the medicinal potential of these newly identified resources.

## 2 Materials and methods

### 2.1 Plant material

In this study, all plant materials were collected from open-field cultivated populations during the flowering period (April–May) of 2018–2019. These cultivated populations originated from wild sources, as local farmers initially obtained seedlings or rhizomes from nearby mountainous regions and subsequently transplanted and propagated them under open-field conditions without environmental control. The cultivation environments closely resembled the species’ native habitats in terms of climate and soil conditions. A total of 26 *Paris* species and varieties were sampled from Hubei, Hunan, Yunnan, Guangxi, Hainan, and Jilin provinces in China. Detailed information on plant names, growth time, location, and specimen codes are provided in Supplementary Table S1. The botanical identification was confirmed by Associate Professor Zhi Wang (Hunan University of Chinese Medicine) based on morphological characteristics, and voucher specimens were deposited at the Yuelushan Laboratory, Hunan Agricultural University (Hunan, China), and the Pharmacophylogeny Centre, Institute of Medicinal Plant Development, Peking Union Medical College (Beijing, China). The collected samples were cleaned, and the rhizomes were dried at 60°C, ground into powder, and passed through a 3-mesh sieve for further analysis.

### 2.2 Chemical and reagents

HPLC-grade methanol and acetonitrile were obtained from Merck (Darmstadt, Germany). Reference standards were purchased from Must Biotechnology Company (Chengdu, China) with purities exceeding 98%. The compounds and their batch numbers were as follows: Polyphyllin I (MUST-19121711), Polyphyllin II (MUST-19071610), Polyphyllin VI (MUST-20052406), Polyphyllin VII (MUST-20051312), Gracillin (MUST-20052811), Prosapogenin A (MUST-19121710), Diosgenin-3-O-Rha (1→2)-[Glc (1→6)]-Glc (MUST-20060101), 17-Hydroxygracillin (MUST-20060103), Dioscin (MUST-20052201), and Pennogenin 3-O-*β*-chacotrioside (MUST-20060102).

### 2.3 Sample preparation

For standard preparation, 16 mg of each reference compound was accurately weighed and dissolved in methanol in 10 mL volumetric flasks to obtain a stock solution of 1.6 mg mL^−1^. The mixed standard solution was then filtered through a 0.22 μm microporous membrane for subsequent analysis. For sample preparation, approximately 0.1 g of dried *Paris* powder was weighed into a 2.0 mL centrifuge tube and extracted with 1 mL of methanol. The mixture was soaked overnight, followed by ultrasonic extraction for 30 min. After cooling to room temperature, the methanol volume was adjusted to the initial level. The extracts were then centrifuged at 12,000 × g for 10 min, and the supernatants were filtered through a 0.22 μm microporous membrane to obtain the test solutions. Quality control (QC) samples were prepared by pooling equal volumes of all test solutions.

### 2.4 UHPLC-Q-TOF/MS analysis

Chromatographic analysis was conducted using an Agilent 1290 Infinity II UHPLC system, coupled with an Agilent 6545 quadrupole time-of-flight mass spectrometer (Q-TOF MS) equipped with a dual AJS electrospray ionization (ESI) source (Agilent Technologies, Santa Clara, CA, United States). Separation was performed on an Agilent ZORBAX SB C18 column (2.1 × 100 mm, 1.8 µm) maintained at 35°C. The mobile phase consisted of solvent A (water with 0.1% formic acid) and solvent B (acetonitrile with 0.1% formic acid), with a flow rate of 0.15 mL/min and an injection volume of 1 µL. The gradient elution program was as follows: 0–1 min, 5% B; 1–5 min, 5%–18% B; 5–15 min, 18%–20% B; 15–23 min, 20%–27% B; 23–35 min, 27%–40% B; 35–42 min, 40%–50% B; 42–45 min, 50%–65% B; 45–46 min, 65%–98% B; 46–60 min, 98% B.

The mass spectrometer was operated in negative ion mode with full-scan acquisition over a mass range of 100–3000 Da in centroid mode. The capillary voltage was set at 4 kV, and the fragmentation voltage at 120 V. The sheath gas temperature and flow rate were 365°C and 11.0 L/min, respectively, while the desolvation gas temperature and flow rate were 325°C and 9.0 L/min. High-purity nitrogen was used as both the nebulizer and collision gas.

### 2.5 Data processing, compound identification and annotation

The raw LC-MS data were converted to mZML format using MS Convert. Metabolomics data preprocessing, including retention time correction, peak detection, peak extraction, peak integration, and peak alignment, was performed using XCMS Online (https://xcmsonline.scripps.edu/) (Forsberg et al., 2018). The maximum tolerated *m/z* deviation was set to 3 ppm, and peaks with a width of 15–25 s and a signal-to-noise ratio greater than 100 were retained. To ensure data reliability, the dataset was normalized using Loess regression, and only ions with relative standard deviations below 40% in QC samples were included for further analysis (Chen L. et al., 2023).

An in-house database comprising 435 compounds was established based on a review of literature related to *Paris* species. This database included detailed information such as compound names, chemical formulas, molecular weights, PubChem IDs, and SMILES codes. LC-MS data analysis was conducted using Agilent MassHunter Qualitative Software (Version B.08.00, Agilent Technologies, United States). Metabolite identification was performed by comparing chromatographic retention time (RT) and ion fragmentation patterns with those of reference standards, as well as utilizing spectral data from databases such as PubChem, MassBank, and Reaxys. By summarizing the chromatographic retention behaviors and ion fragmentation rules of various steroidal saponins, this approach facilitated the comprehensive characterization of the chemical profile of *Paris* species.

### 2.6 Metabolic profile analysis

Multivariate statistical analyses, including HCA, PCA, and OPLS-DA, were conducted using SIMCA 14.1 (Umetrics AB, Umeå, Sweden). To prevent overfitting, a permutation test with 200 iterations was performed. Key variables contributing to group separation were identified based on *p*-values and Variable Importance in Projection (VIP) scores from OPLS-DA. Metabolites with *p* < 0.05 and VIP >1.8 were considered statistically significant and classified as differentially accumulated metabolites (DAMs). Data visualization, including metabolic profile plots, Pearson correlation analysis, and bar charts, was carried out using Origin 2021, while clustering heatmaps were generated with TBtools (Chen C. et al., 2023).

## 3 Results

### 3.1 Mass spectral fragmentation and chromatographic retention behavior of steroidal saponins in *Paris*

**FIGURE 1:**
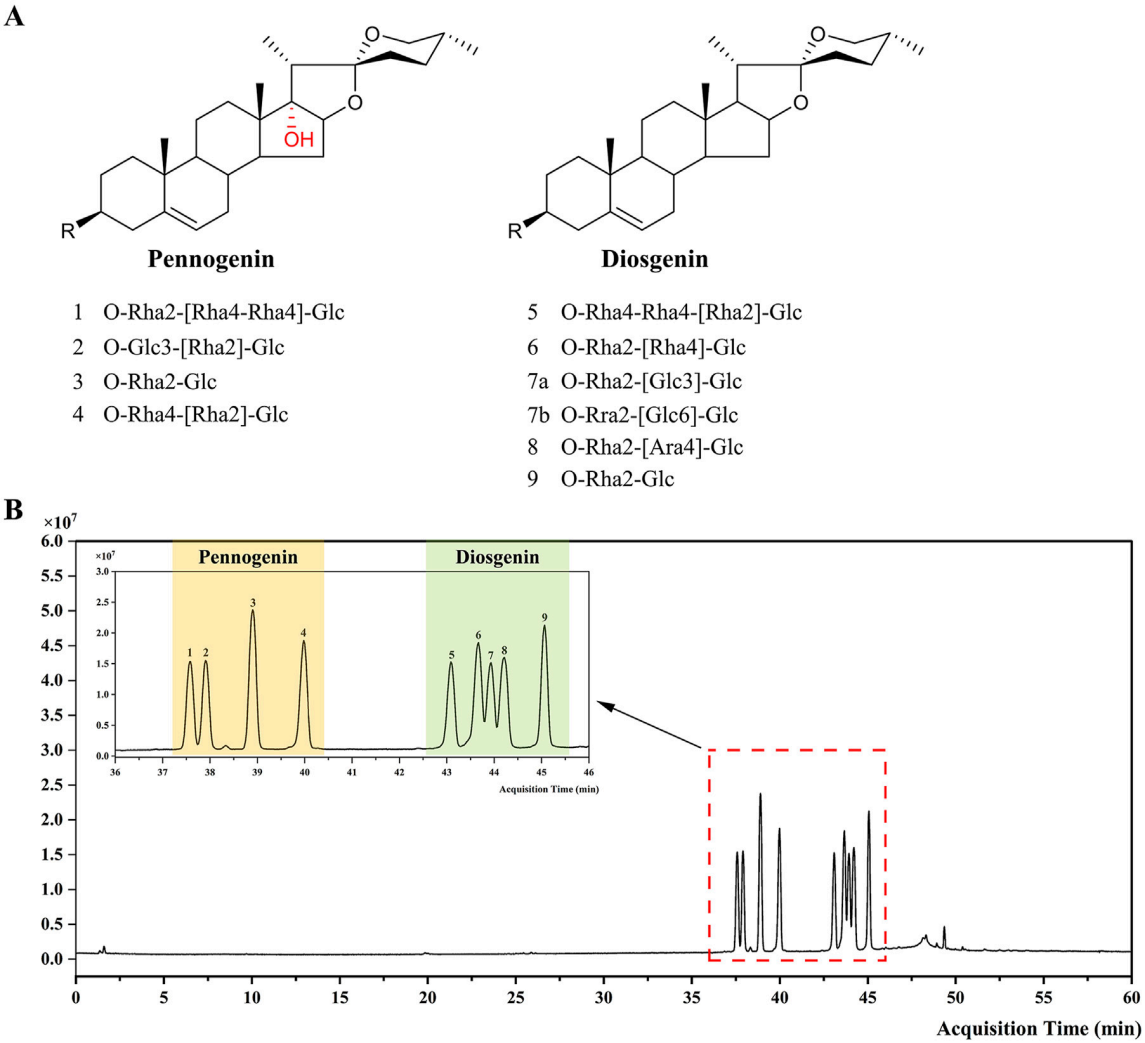
Chemical structures of steroidal saponin standards and their total ion chromatogram. **(A)** Chemical structures of 10 steroidal saponin reference standards, with compounds **4**, **7a**, and **7b** are isomers. **(B)** Total ion chromatogram of the mixed standard solution obtained via LC-MS analysis. Peaks 1–4 correspond to pennogenin saponins (yellow), with RTs between 37.34 and 40.38 min. Peaks 5–9 correspond to diosgenin saponins (green), with RTs between 42.79 and 45.39 min. **1**. Polyphyllin VII; **2**. 17-Hydroxygracillin; **3**. Polyphyllin VI; **4**. Pennogenin 3-O-*β*-chacotrioside; **5**. Polyphyllin II; **6**. Dioscin; **7**. Gracillin/Diosgenin-3-O-Rha (1→2)-[Glc (1→6)]-Glc; **8**. Polyphyllin I; **9**. Prosapogenin A.

Steroidal saponins exhibit structural diversity, with a sapogenin core attached to one or more sugar residues. Based on the aglycone structure, these compounds are classified into diosgenin-type, pennogenin-type, furostanol-type saponins, and polyhydroxy glycosides. The sugar moieties primarily include *D*-glucose (Glc), *L*-rhamnose (Rha), and *L*-arabinofuranose (Ara), with minor contributions from *D*-xylose (Xyl), fucose, and *D*-apiose (Api) (Ding et al., 2021). To enhance compound identification accuracy, this study summarized characteristic fragmentation patterns and chromatographic retention behaviors of steroidal saponins from the literature and further verified them using ten reference standards, including four pennogenin saponins and six disogenin saponins (Figure 1A).

The molecular mass and formula can typically be obtained from the [M+Cl]^−^, [M+HCOO]^−^ and [M–H]^−^ ions. The classes and number of sugar units can be inferred from the loss of neutral fragments such as 162 Da (Glc/Gal), 146 Da (Rha/Fuc), and 132 Da (Ara/Xyl). Successive sugar losses are observed in negative MS/MS mode (Liang et al., 2019). From the ESI(−) data, the total ion chromatogram (TIC) of the 10 reference standards (Figure 1B) revealed nine distinct peaks, with detailed identification results provided in Supplementary Table S2. Mass spectra predominantly exhibited [M+Cl]^−^ and [M+HCOO]^−^ adduct ion peaks, alongside a weaker [M–H]^−^ quasi-molecular ion peak (Zhang T. et al., 2010). An unknown [M+93]^−^ adduct ion was also observed.

Although mass spectrometry provides valuable information, such as the molecular weight of compounds, it cannot distinguish between isomers. Chromatographic RT serves as an additional identifier for distinguishing compound types. Peaks **1–4** corresponded to pennogenin saponins (RT: 37.34–40.38 min), and peaks **5–9** were identified as diosgenin saponins (RT: 42.79–45.39 min). Notably, Pennogenin 3-O-*β*-chacotrioside (**4**), Gracillin **(7a)**, and Diosgenin-3-O-Rha (1→2)-[Glc (1→6)]-Glc **(7b)** were found to be isomers. Based on the different RTs of pennogenin saponins and diosgenin saponins, Peak **4** was identified as Pennogenin 3-O-*β*-chacotrioside, a pennogenin saponin, while Peak **7** corresponded to Gracillin and Diosgenin-3-O-Rha (1→2)-[Glc (1→6)]-Glc, two diosgenin saponins that differ only in the positioning of their sugar moieties. Therefore, Peak 7 was tentatively assigned as a common peak for these two compounds.

Based on UHPLC-Q-TOF/MS data and prior studies (Huang, 2019), the chromatographic retention order of steroidal saponins was summarized as follows: polyhydroxy glycosides < furostanol-type saponins < pennogenin-type saponins < diosgenin-type saponins. The RT of steroidal saponins on a reverse-phase column is influenced by several structural factors:

1. Number and position of hydroxyl groups on the aglycone: A higher number of hydroxyl groups results in shorter RTs. For instance, polyhydroxylated spirostanols exhibit shorter RTs than furostanol-type compounds due to the increased polarity.
2. F-Ring configuration: Furostanol-type saponins with an open F-ring elute earlier than spirostanol-type saponins (closed F-ring).
3. C-17 substitution: Pennogenin saponins possessing a hydroxyl group at C-17 exhibit shorter RTs compared to diosgenin saponins, which lack this group.
4. Sugar chain length and composition: Among saponins with the same aglycone, longer sugar chains result in shorter RTs. Additionally, for sugar chains of equal length, RTs decrease in the order of Rha < Glc < Ara.

### 3.2 Metabolic diversity and chemotaxonomic profiling of *Paris* species

#### 3.2.1 PCA and HCA analysis

**FIGURE 2:**
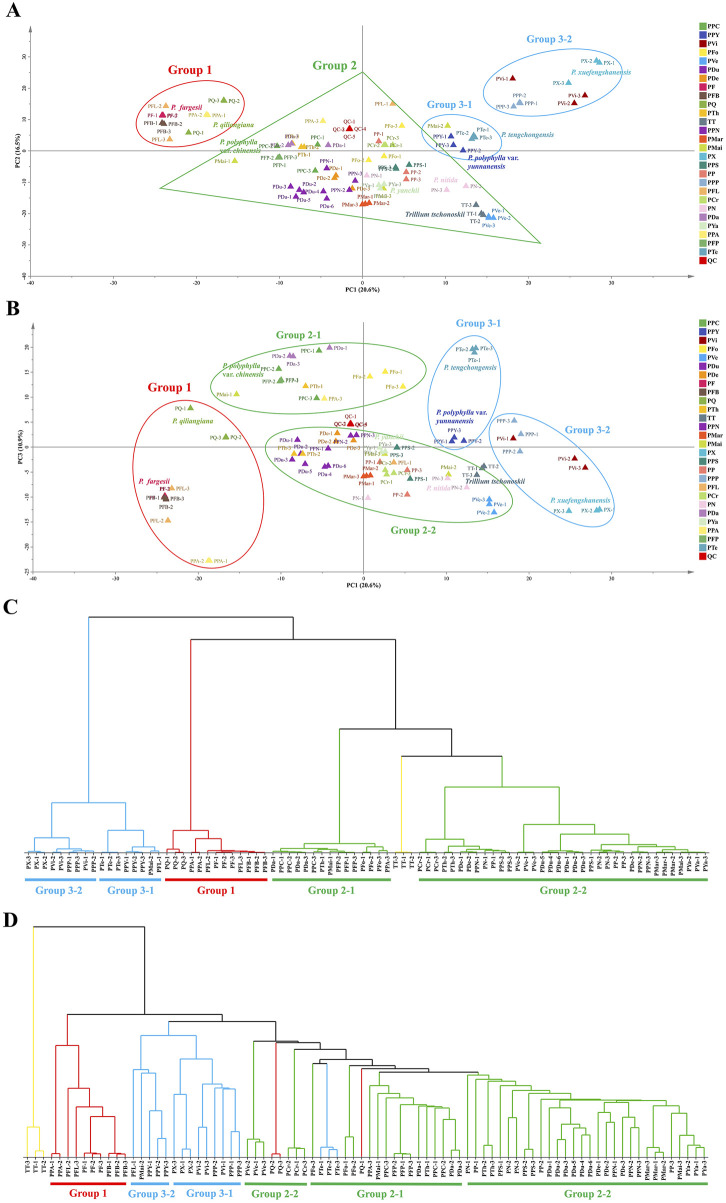
Metabolomics-based classification of *Paris* species using PCA and HCA. **(A,B)** PCA score plots. Panel A shows a PCA score plot of PC1 (20.6%) vs. PC2 (16.5%), and Panel B shows PC1 (20.6%) vs. PC3 (10.9%). QC samples (red triangles) cluster at the center, confirming data reliability. Samples are classified into three main metabolic groups. **(C,D)** HCA dendrograms. Panel C shows the dendrogram based on Ward linkage, while Panel D is based on Single linkage. Both methods largely align with PCA results, though discrepancies exist, such as in the classification of *P. qiliangiana* and *P. tengchongensis*.

After preprocessing the non-targeted metabolomics data, a total of 1,110 metabolite characteristic variables were obtained, including sample name, RT, *m/z* value, and normalized peak intensity. PCA and HCA were performed to explore the metabolic patterns across different samples and assess their correlations (Figure 2). The first three principal components collectively explained 48% of the total variance (PC1 = 20.6%, PC2 = 16.5%, and PC3 = 10.9%). Figures 2A,B show the PCA score plots of PC1 versus PC2 and PC1 versus PC3, respectively, where the proximity of sample points reflects the similarity of their metabolic profiles. QC samples, marked by red triangles, clustered tightly at the center of the PCA plots, confirming the stability of the analytical platform and ensuring that the observed metabolic differences were attributable to biological variation rather than instrumental or environmental fluctuations. Figures 2C,D present HCA dendrograms constructed using the Ward linkage and single linkage methods, respectively.

Based on the combined results of PCA and HCA, the samples were classified into three major groups. Group 1, predominantly located in the second quadrant, mainly comprises *P. fargesii* (PF) and its variants. Group 2, which contains the largest number of samples, spans all four quadrants in Figure 2A. According to the HCA results using the Ward linkage method (Figure 2C), this group can be further divided into two subgroups, which is consistent with the distribution in the PC1 versus PC3 score plot (Figure 2B). Group 3 tends to split into two subsets, as further validated by the corresponding branches in the HCA dendrograms (Figures 2C,D).

Additionally, the classifications of *P. qiliangiana* (PQ) and *P. tengchongensis* (PTe) differed between the two HCA methods: in Figure 2C, PQ was assigned to Group 1 and PTe to Group 3-1, whereas in Figure 2D, both were placed in Group 2. Combined with the PCA score plot distribution, the clustering results in Figure 2C are considered more biologically meaningful. Notably, both PCA and HCA analyses revealed that biological replicates of certain species did not cluster tightly. For example, replicates of *P. mairei* (PMai), *P. fargesii* var. *latipetala* (PFL), and *P. polyphylla* var. *appendiculata* (PPA) were relatively dispersed. This variation may be attributed to environmental influences on metabolic profiles, thus posing a challenge to understanding the metabolic diversity of different species of the genus.

#### 3.2.2 Comparative metabolic profiling of different *Paris* groups

**FIGURE 3:**
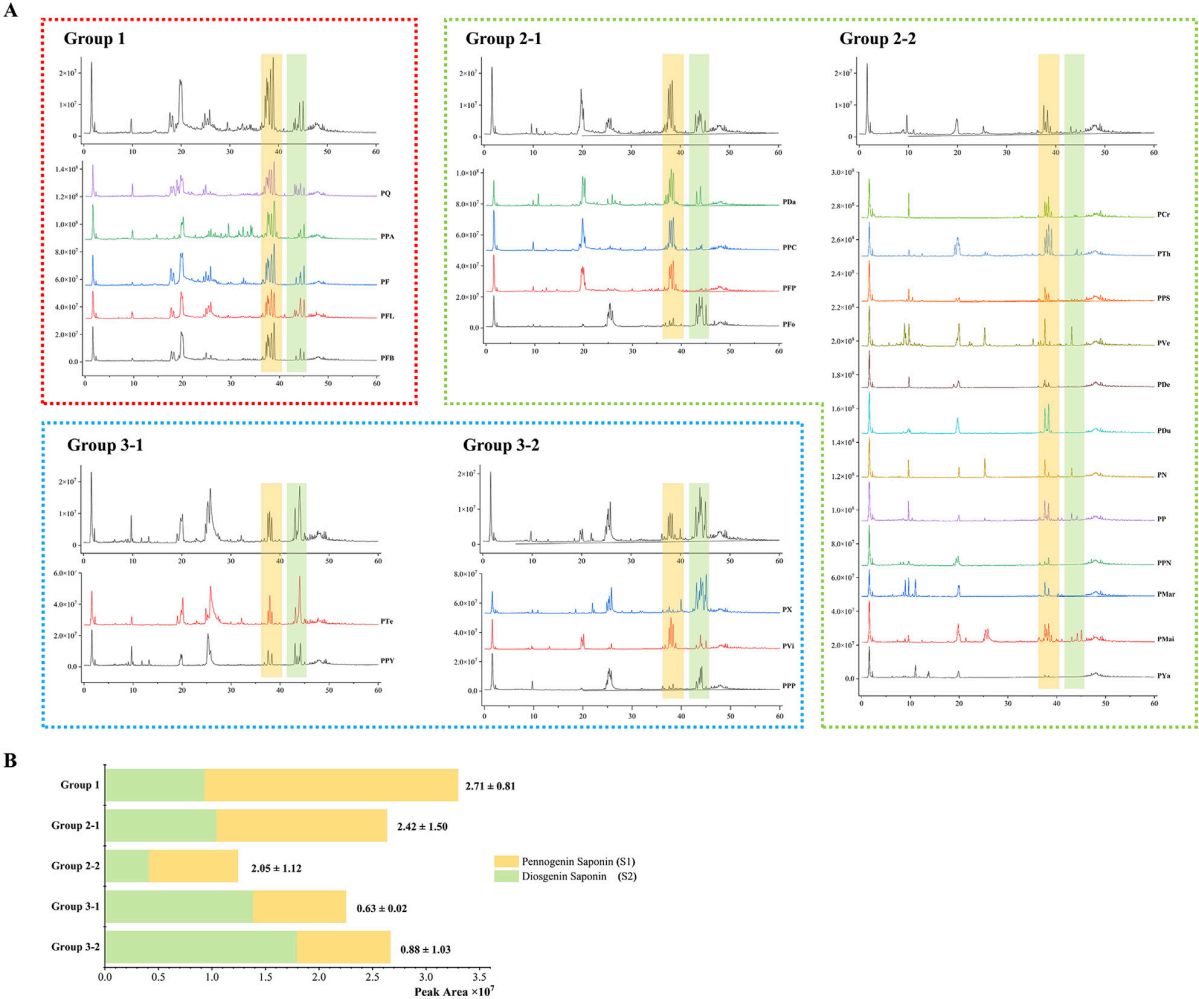
Metabolic profiles and relative distribution of steroidal saponins in different *Paris* groups. **(A)** Total ion chromatograms of *Paris* species across different metabolic groups. The yellow-shaded region (37.34–40.38 min) represents pennogenin saponins (S1), while the green-shaded region (42.79–45.39 min) indicates diosgenin saponins (S2). Variations in saponin composition among groups are evident. **(B)** Stacked bar chart comparing the relative peak areas of S1 and S2. The average S1/S2 ratio ±SD is shown above each bar. Group 1 has the highest S1 content (S1/S2 = 2.71 ± 0.81), while Group 3 is dominated by S2. Group 1 exhibits the highest S1 content (S1/S2 = 2.71 ± 0.81), whereas Group 3 is predominantly composed of S2. Statistically significant differences in S1 and S2 levels are observed in Group 1 and Group 2-2 (*p* < 0.05), while variations in Groups 2-1, 3-1, and 3-2 are not statistically significant (*p* ≥ 0.05).

Based on UHPLC-Q-TOF/MS non-targeted metabolomics data, substantial metabolic differences were observed in different *Paris* groups (Figure 3). Group 1 displayed a unique metabolic profile, with prominent peaks in the 15–50 min RT range, indicating the presence of both pennogenin and diosgenin saponins, along with spirostanol and furostanol metabolites. Group 2 exhibited the highest metabolic variability, with two subgroups (Group 2-1 and Group 2-2) showing distinct differences in steroidal saponin content. Group 3 was characterized by several prominent peaks in the 20–30 min RT range, with a higher abundance of diosgenin saponins than pennogenin saponins. These findings underscore the metabolic diversity within *Paris* species, revealing distinct biochemical compositions that may contribute to their functional differentiation.

To further elucidate the metabolic diversity of *Paris* groups, the distribution patterns of pennogenin and diosgenin saponins—the main bioactive components of Rhizoma Paridis, were analyzed by integrating the findings from Section 3.1. The summed peak areas of these two saponin types were extracted and labeled as S1 (pennogenin saponins) and S2 (diosgenin saponins), revealing significant variation across groups.

In Group 1, pennogenin saponins were predominant, with a significantly higher content than diosgenin saponins (S1/S2 = 2.71 ± 0.81, *p* < 0.05), indicating that the metabolic profile of this group is biased toward pennogenin saponin enrichment.

Group 2, which contained the largest number of samples, exhibited subgroup-specific differences in S1 and S2 composition. Within Group 2-1, the overall abundance of pennogenin saponins exceeded that of diosgenin saponins (S1/S2 = 2.42 ± 1.50). This subgroup includes PPY, *P. daliensis* (PDa), *P. fargesii* var. *petiolate* (PFP), and *P. forrestii* (PFo), among which PPY, PDa, and PFP were characterized by a higher accumulation of pennogenin saponins, whereas PFo was metabolically distinct due to its greater abundance of diosgenin saponins. Conversely, Group 2-2 exhibited lower overall steroidal saponin content but remained enriched in pennogenin saponins (S1/S2 = 2.05 ± 1.12, *p* < 0.05). Species such as *P. cronquistii* (PCr), *P. thibetica* (PTh), *P. dunniana* (PDu), and *P. polyphylla* (PP) were representatives of this enrichment pattern.

In Group 3, diosgenin saponins were more abundant than pennogenin saponins. Within this group, Group 3-1, consisting of PTe and PPY, exhibited a similar metabolic profile, characterized by a higher proportion of other steroidal saponins. Group 3-2, which included *P. xuefengshanensis* (PX), *P. vietnamensis* (PVi), and *P. polyphylla* var. *pseudothibetica* (PPP), was characterized by a significant enrichment of diosgenin saponins (S1/S2 = 0.88 ± 1.03). PX, in particular, exhibited both a high peak area and a wide diversity of diosgenin saponins, making it a representative species within this subgroup. Interestingly, PVi in Group 3-2 displayed a higher abundance of pennogenin saponins, which was an unexpected finding, suggesting the presence of metabolic diversity even within seemingly homogenous groups.

### 3.3 Metabolic characteristics of newly identified *Paris* species

This study investigated five *Paris* species newly discovered during the fourth national census of Chinese medicine resources, all published after 2017: *P. nitida* (PN) (Wang Z. et al., 2017), *P. qiliangiana* (PQ) (Yang et al., 2017), *P. xuefengshanensis* (PX) (Wang et al., 2024), *P. yanchii* (PYa) (Li et al., 2017), and *P. tengchongensis* (PTe) (Ji et al., 2017). To assess their metabolic profiles and medicinal potential, a comparative metabolomic analysis was conducted alongside the official medicinal species PPC and PPY. This analysis identified both common metabolites and DAMs across the seven species, providing insights into their metabolic relationships.

#### 3.3.1 Identification of common metabolites

**FIGURE 4:**
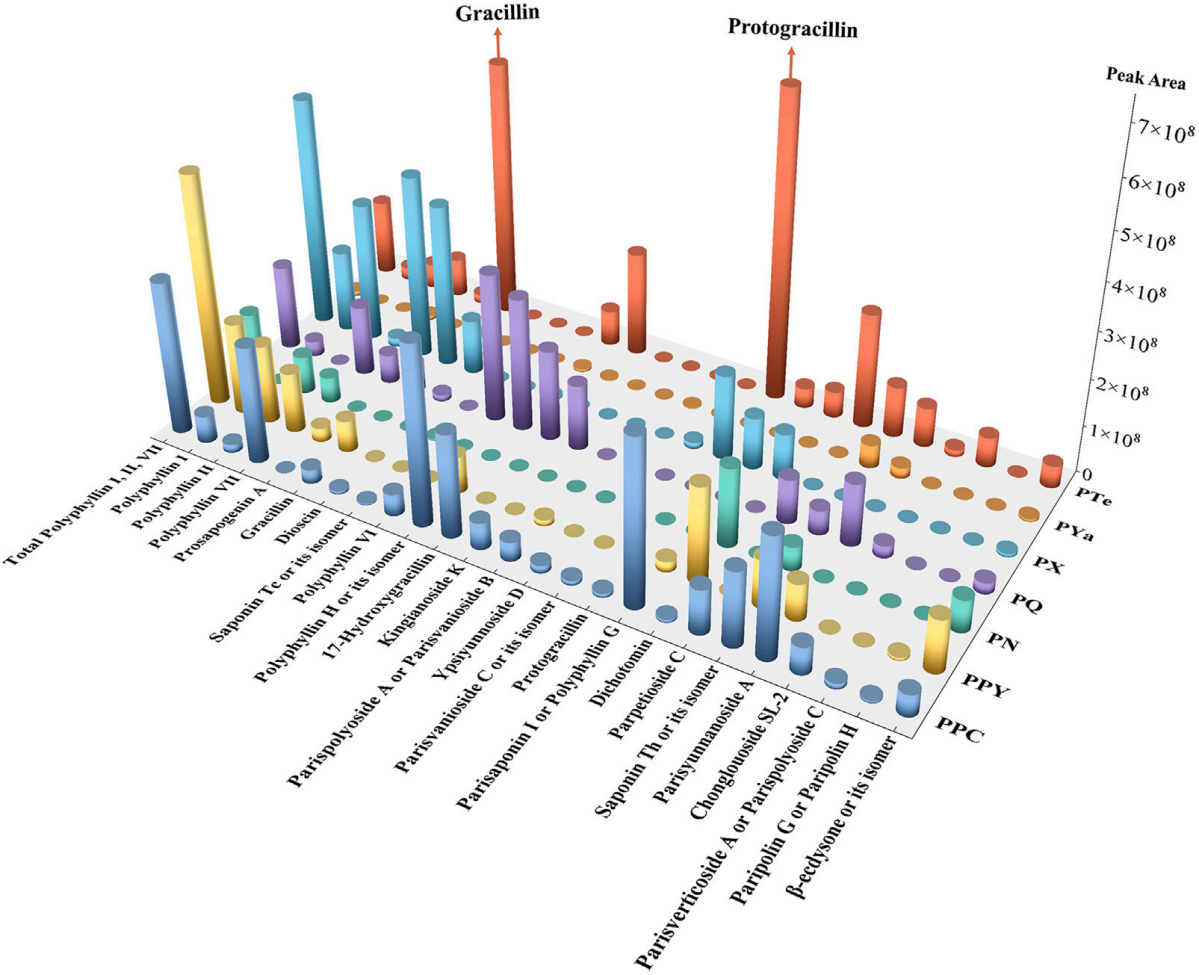
Relative abundance of common metabolites in seven *Paris* Species. A three-dimensional bar chart depicting the relative abundance of 24 common metabolites across seven *Paris* species (PPC, PPY, PN, PQ, PX, PYa, and PTe). Bar height indicates metabolite abundance variations among species. Notably, the total contents of Polyphyllin I, II, and VII are relatively high in PX and PQ, with PX exceeding PPC and PPY. Additionally, PTe exhibits significantly elevated levels of Gracillin and Protogracillin, highlighting its potential medicinal value.

Common metabolites play essential roles in metabolic pathways and pharmacological activities. In this study, pairwise comparisons were conducted between each of the five newly identified species and both PPC and PPY, along with an additional comparison between PPC and PPY. In each comparison group, shared peaks with a relative peak area ≥10% in the TIC were identified as common metabolites (Li et al., 2024). A total of 24 common metabolites were identified (Supplementary Table S3). Based on the average peak area within each species, a three-dimensional bar chart was generated (Figure 4). Among these metabolites, Polyphyllin VII and *β*-ecdysone were present in all seven species. Polyphyllin VII is the only pennogenin saponin specified in the Chinese Pharmacopoeia for the quantitative determination of Rhizoma Paridis (Xiang et al., 2023).

The total content of Polyphyllin I, II, and VII followed the order: PX > PPY > PPC > PQ > PTe > PN > PYa. PX exhibited an exceptionally high level of these compounds, surpassing even PPY and PPC, while PQ also showed relatively high concentrations. Notably, PTe displayed elevated levels of Gracillin (a diosgenin saponin) and its precursor, Protogracillin, suggesting its potential as a resource for Rhizoma Paridis. In contrast, PN and PYa contained relatively low total saponin content, which may not meet pharmacopoeial standards.

#### 3.3.2 Screening and identification of DAMs

Through supervised OPLS-DA analysis (Supplementary Figure S1), a total of 43 DAMs (P1-P43) were identified based on VIP >1.8 and *p* < 0.05 (Supplementary Table S4). These DAMs included 12 furostanol-type, 11 spirostanol-type, 18 isospirostanol-type (comprising 4 pennogenin-type and 7 diosgenin-type), and 2 cholestanol-type saponins.

**FIGURE 5:**
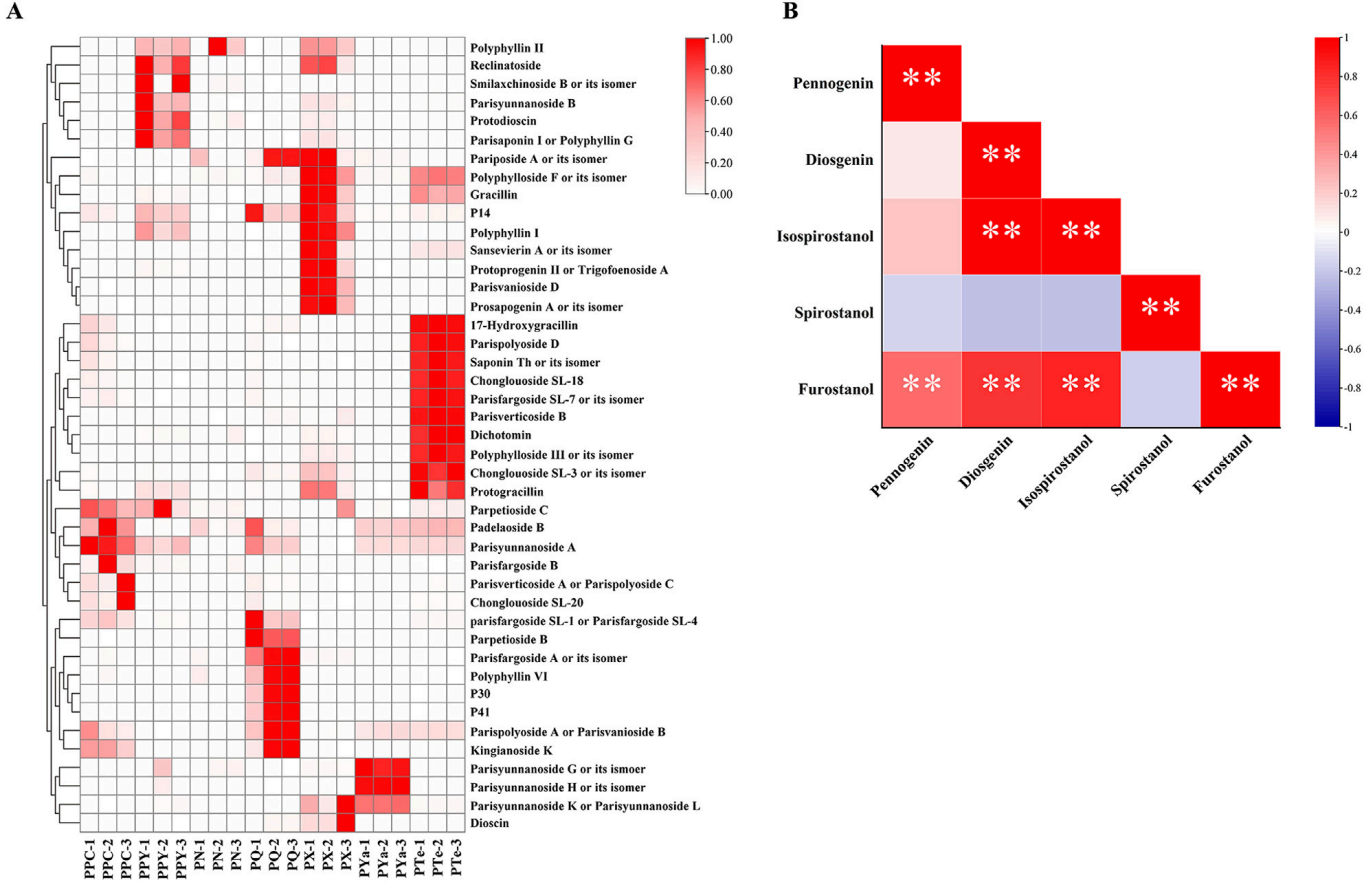
Heatmap and correlation analysis of differentially accumulated metabolites in seven *Paris* species. **(A)** Heatmap displaying the relative abundance of DAMs (normalized 0–1) across seven *Paris* species. Darker red shading indicates higher metabolite expression levels. Hierarchical clustering reveals distinct groups of metabolites with similar accumulation patterns. **(B)** Pearson correlation heatmap of steroidal saponins. Red indicates a positive correlation, while blue represents a negative correlation. Asterisks (*) denote statistical significance (*p < 0.05, **p < 0.01). Notably, furostanol-type and isospirostanol-type saponins exhibit a strong positive correlation, whereas spirostanol-type saponins show a negative correlation with other types, though this is not statistically significant.

To further examine the distribution and interrelationships of DAMs, clustering analysis was performed using ion intensity data, and the results were visualized in a heatmap (Figure 5A). The analysis revealed species-specific metabolite profiles, suggesting potential Q-markers for Rhizoma Paridis quality control (Wang et al., 2022; Li et al., 2024). For instance, PPC exhibited high levels of Parpetioside C, Padelaoside B, and Parisyunnanoside A, while PPY was enriched in Polyphyllin II, Reclinatoside, Smilaxchinoside B, Parisyunnanoside B, Protodioscin, and Parisaponin I. Among the newly identified species, PYa was characterized by Parisyunnanoside G, H, and K/L, all spirostanol-type compounds; PQ exhibited elevated levels of Parpetioside B, Parisfargoside A, and Polyphyllin VI; PX showed higher levels of Pariposide A, Polyphylloside F, and Polyphyllin I; and PTe was distinguished by significantly higher levels of 17-Hydroxygracillin, Parispolyoside D, and Saponin Th.

Steroidal saponins are the primary bioactive compounds in *Paris*. In this study, they were categorized into isospirostanol-type (including diosgenin and pennogenin saponins), spirostanol-type, and furostanol-type saponins based on their structural characteristics (Ding et al., 2021). To assess the consistency of metabolite variation trends, Pearson correlation analysis was conducted on different types of steroidal saponins (Figure 5B). A highly significant positive correlation (*p* < 0.01) was observed between furostanol-type and isospirostanol-type saponins, suggesting a coordinated accumulation of these metabolites. Conversely, spirostanol-type saponins exhibited a negative correlation with the other steroidal saponin types, though this relationship was not statistically significant (*p* > 0.05). Previous studies have indicated that furostanol-type steroidal saponins are not entirely retained in plants. Instead, some convert into more bioactive spirostanol or isospirostanol saponins under specific conditions, such as enzymatic catalysis, high-temperature treatment, or storage environment (Guan et al., 2021; Jin et al., 2023; Yuan et al., 2022). The present result suggests that furostanol saponins may preferentially convert into isospirostanol saponins rather than spirostanol saponins. However, this transformation mechanism remains largely unexplored, necessitating further investigation.

## 4 Discussion

This study provides a comprehensive metabolic analysis of *Paris* species. PCA and HCA classified 26 *Paris* species and varieties into three distinct groups based on their metabolic profiles. Further characterization revealed significant variations in the composition and abundance of pennogenin and diosgenin saponins among these groups. The difference may play a key role in shaping the phylogenetic relationships within *Paris* and offer a chemical basis for understanding its systematics. To further explore the metabolic characteristics of the recently described species, five newly identified *Paris* species were compared with PPC and PPY. The identification of common metabolites highlighted promising new resources with high levels of steroidal saponins, which could serve as candidates for clinical applications and drug development. Clustering and correlation analyses of DAMs enabled the identification of species-specific marker metabolites and provided insights into the metabolic relationships among different types of saponins. These findings contribute to a deeper understanding of the chemical diversity within *Paris* and its potential medicinal value.

While this study revealed major interspecific metabolic differences in *Paris*, several limitations remain, including restricted intraspecific sampling, the lack of absolute quantification, and tentative identification of isomeric compounds. Future research will broaden geographic sampling to better capture intraspecific variation, implement targeted quantitative analyses for key metabolites, and incorporate ion mobility mass spectrometry (IM-MS), which provides an additional separation dimension based on gas-phase ion mobility (Giuseppe et al., 2022), to enhance structural resolution and improve the confident identification of isomers.

### 4.1 Metabolic patterns and their implications for *Paris* classification

The classification of the genus *Paris* has long been debated, with discrepancies between morphological and phylogenetic classifications (Li, 1998; Ji et al., 2006; 2019; 2020). Li Heng classified the genus into two subgenera based on placental type, Subgenus *Daiswa* H. Li and Subgenus *Paris* H. Li, further dividing them into eight groups (Li, 1998). In contrast, Ji et al. proposed that *Paris* is a monophyletic genus and classified it into five groups based on phylogenetic analysis (Ji, 2021). In this study, metabolomic analysis of 26 *Paris* species and varieties partially aligned with existing classification patterns and provided insights into the taxonomy of controversial species.

Based on metabolic profiles, all samples were classified into three major groups. In Group 1, the metabolic similarity among PF and its variants supports Ji’s classification of PFL and PFB as synonyms of PF (Ji, 2021). Group 2, the largest cluster, was further divided into two subgroups. Group 2-1 comprised species from both Sect. *Euthyra* (PDa, PPC, PFP) and Sect. *Axiparis* (PFo). Notably, PFo exhibited significant metabolic divergence from the other species in this subgroup, as reflected by its distinct positioning in the first quadrant of Figure 2B. Group 3 was also divided into two subgroups. Group 3-1 included PTe and PPY. Although PTe has been classified in Sect. *Axiparis* based on morphological (Ji et al., 2017) and chloroplast phylogenetic evidence (Ji et al., 2019), its close clustering with PPY in the PCA score plot suggests highly similar metabolic profiles, warranting further investigation into its classification. In Group 3-2, PX, PVi, and PPP clustered together. While PVi and PPP belong to Sect. *Euthyra*, PX has a distinct placental structure, further complicating its taxonomic placement (Wang et al., 2024).

Metabolomic analysis provided valuable insights into the classification of certain controversial species. First, the taxonomic status of PFP remains unresolved. Li Heng considered PFP a synonym of *P. delavayi* var. *petiolata* (Baker ex C. H. Wright) Li (1998), whereas Ji classified it as a synonym of *P. delavayi* (PDe) (Ji, 2021). Ren et al. reported significant differences in phenology and saponin content between PFP and PF, suggesting that PFP should be recognized as a variety of PDe (Ren et al., 2023). In this study, PFP clustered within Group 2-1, yet its metabolic profile, dominated by pennogenin saponins, closely resembled that of PF and its variants in Group 1. In contrast, PDe was placed in Group 2-2, exhibiting markedly different metabolic traits. These findings challenge the classification of PFP as a variety of PDe. Second, the taxonomic status of PP and its varieties has been debated. Takhtajan elevated its varieties (PPC, PPY) to independent species based on morphological distinctions (Takhtajan, 1983), whereas Li Heng argued that these traits are highly variable and insufficient for species differentiation (Li, 1998). Phylogenetic analyses have failed to support a monophyletic PP complex and its varieties, leading Yang to reclassify its varieties—PPC, PPY, and *P. polyphylla* var. *stenophylla* (PPS) as independent species (Yang, 2020; Ji, 2021). Metabolomic data partially support this view. PP and PPS displayed similar metabolic profiles within Group 2-2, while PPC and PPY were classified into Group 2-1 and Group 3-1, respectively, showing substantial metabolic differences from PP and PPS. These findings support the recognition of PPC and PPY as distinct species.

PCA and metabolic profiling indicated that the concentrations of pennogenin and diosgenin saponins are key factors distinguishing metabolic traits among *Paris* species. A clear bifurcation was observed: species with higher pennogenin saponin content were primarily positioned in the second and third quadrants of the PCA score plot, corresponding to Group 1 and Group 2-1 (except PFo). In contrast, species with higher diosgenin saponin content clustered in the first and fourth quadrants, including Group 3 and PFo (Figures 2A,B). An exception to this trend was PVi, which belongs to Group 3 but predominantly contains pennogenin saponins. These findings align with previous studies on steroidal saponin content (Chen, 2021; Yu, 2022). Additionally, species in Group 2-2, such as *P. polyphylla* var. *nana* (PPN), PPS, PDe, PN, and PYa, exhibited significantly lower total saponin levels and clustered near the center of the PCA score plot. This further supports the conclusion that pennogenin and diosgenin saponins are key determinants of the metabolic classification of *Paris* species.

### 4.2 Metabolomic insights into the medicinal potential of newly identified *Paris* species

Five newly identified *Paris* species, PN, PQ, PX, PYa, and PTe, have not been extensively studied regarding their chemical composition and pharmacological properties. This study employed a comprehensive metabolomic analysis to evaluate their medicinal potential and provide a foundation for future pharmacological investigations.

PX, a recently published species (Wang et al., 2024), is believed to contain structurally diverse compounds with strong anticancer and antimicrobial properties (Yang et al., 2024b). Our metabolomic analysis and preliminary mass spectrometric identification revealed a high abundance of steroidal saponins, predominantly of the diosgenin type, with some potentially novel compounds. Notably, the total content of Polyphyllin I, II, and VII in PX exceeds that of PPY and PPC, highlighting its potential as a valuable medicinal resource. PQ has a relatively wide distribution in western China (Yang et al., 2017), contains high levels of saponins (Wang et al., 2018; Liu B. et al., 2019), and demonstrates antibacterial and antioxidant activities (He et al., 2022; Sun et al., 2022). Our analysis confirmed its enrichment in pennogenin saponins, with notably high levels of Polyphyllin I, II, and VII, further underscoring its medicinal significance. PTe exhibited a metabolic profile nearly identical to that of PPY. Previous studies have identified common components between these two species (Wang et al., 2020; Sun et al., 2024) and demonstrated notable antifungal activity in PTe (Yang et al., 2024a). Quantitative analysis indicated that its steroidal saponin content exceeds pharmacopoeial standards (Liu, 2021). Furthermore, our study revealed that PTe is particularly enriched in Gracillin and its precursor, Protogracillin. Protogracillin undergoes enzymatic conversion to Gracillin through furostanol glycoside 26-O-*β*-glucosidase (F26G) catalysis (Inoue et al., 1996). Gracillin has been shown to induce apoptosis, thereby inhibiting multiple cancer cell lines (Min et al., 2019). These findings suggest that PTe possesses strong antitumor potential, warranting further investigation.

In contrast, research on PN and PYa remains limited. Previous studies have reported extremely low saponin content in PN (Yin et al., 2020). Consistently, our analysis revealed that both PN and PYa contain low levels of steroidal saponins and lack characteristic metabolites, suggesting that they may not be suitable for medicinal use. Instead, conservation efforts should be prioritized to preserve the biodiversity of *Paris* species.

The biological activities of pennogenin and diosgenin saponins differ significantly. Diosgenin saponins, such as Polyphyllin I (Yang R. Y. et al., 2024), Polyphyllin II (Chen et al., 2019), Polyphyllin D (Cheung et al., 2005), and Dioscin (Zhou et al., 2021), exhibit strong antitumor activity. In contrast, hydroxylation at the C-17 position reduces the antitumor efficacy of pennogenin saponins (Zhao et al., 2009; Ding et al., 2021), which primarily exert hemostatic effects and are considered key contributors to the hemostatic properties of *Paris* species (Fu et al., 2008; Wang et al., 2013). Examples include Polyphyllin VII (Tg) (Cong et al., 2012) and Paris Saponin H (Wen et al., 2019). Consistent with previous findings (Wen et al., 2023), our analysis confirmed that PPC is richer in pennogenin saponins, whereas PPY primarily contains diosgenin saponins. As a result, PPC may exhibit stronger hemostatic activity, while PPY shows greater potential for antitumor effects (Li, 2012; Ding et al., 2017).

The relative proportions of pennogenin and diosgenin saponins serve as key indicators of pharmacological tendencies, particularly related to antitumor and hemostatic activities among different *Paris* species. In this study, species with an S1/S2 ratio <1 are speculated to exhibit antitumor activity, whereas those with S1/S2 ≥1 are more likely to possess pronounced hemostatic effects. Based on this hypothesis, the findings suggest that PX and PTe, with their high diosgenin saponin content, are promising candidates for antitumor drug development. In contrast, PQ, which is enriched in pennogenin saponins, may be more suitable for hemostatic applications (Liu et al., 2012). Similarly, PN and PYa exhibit a higher proportion of pennogenin saponins than diosgenin saponins, indicating potential hemostatic activity. However, their relatively low overall saponin content may result in weaker pharmacological potency. Despite these differences, Traditional Chinese Medicine emphasizes the holistic efficacy of medicinal herbs and describes their therapeutic properties using distinct conceptual frameworks (Zhang A. et al., 2010). In the official pharmacopoeia, PPC and PPY are both recognized as source plants for Rhizoma Paridis, with no formal distinction in their efficacy or therapeutic applications, which is based on the combined pharmacological effects of a common class of steroidal saponins, despite variations in the specific proportions of different saponin subtypes. The present study further reveals that the five newly identified *Paris* species contain steroidal saponins to varying degrees. Given that different *Paris* species have historically been used interchangeably in traditional medicine without strict differentiation in efficacy (Liu Y. Y. et al., 2019), these newly identified species are likely to exhibit similar therapeutic effects to PPY and PPC. Specifically, they may possess the ability to clear heat, remove toxins, disperse swelling, relieve pain, calm the liver, and settle convulsions, and thus be applicable for the treatment of carbuncles and abscesses, sore throat, snake and insect bites, traumatic injuries, and convulsions (China, 2020). Overall, the metabolomic analysis conducted in this study provides valuable insights to guide future pharmacological research and clinical applications of *Paris* species. From the perspective of pharmacophylogeny, closely related species or genera exhibit correlations between phylogeny, chemical constituents, and therapeutic effects, the medicinal potential of the five newly identified species is summarized in Table 1.

**TABLE 1:** Pharmacophylogenetic relationships of five newly identified *Paris* species and two official medicinal species.

Plant phylogenetic relationships		Chemical constituents		Efficacy	
Source	Species	Group	S1/S2	Pharmacological activity tendency	Traditional efficacy
Official medicinal species	*P. polyphylla* var. *chinensis* (PPC)	2-1	3.54	Hemostatic	Clear heat, remove toxins, disperse swelling, relieve pain, calm the liver, and settle convulsion
	*P. polyphylla* var. *yunnanensis* (PPY)	3-1	0.65	Antitumor	
Newly identified *Paris* species	*P. xuefengshanensis* (PX)	3-2	0.20	Antitumor	Presumed similar pharmacological effects to PPC and PPY, based on phylogenetic relationships and chemical constituents
	*P. tengchongensis* (PTe)	3-1	0.62	Antitumor	
	*P. qiliangiana* (PQ)	1	2.38	Hemostatic	
	*P. nitida* (PN)	2-2	1.29	Hemostatic	
	*P. yanchii* (PYa)	2-2	1.00	Hemostatic	

Note: The S1/S2 ratio represents the total relative content ratio of pennogenin to diosgenin saponins.

### 4.3 Metabolomics-guided synthetic biology in *Paris* species

**FIGURE 6:**
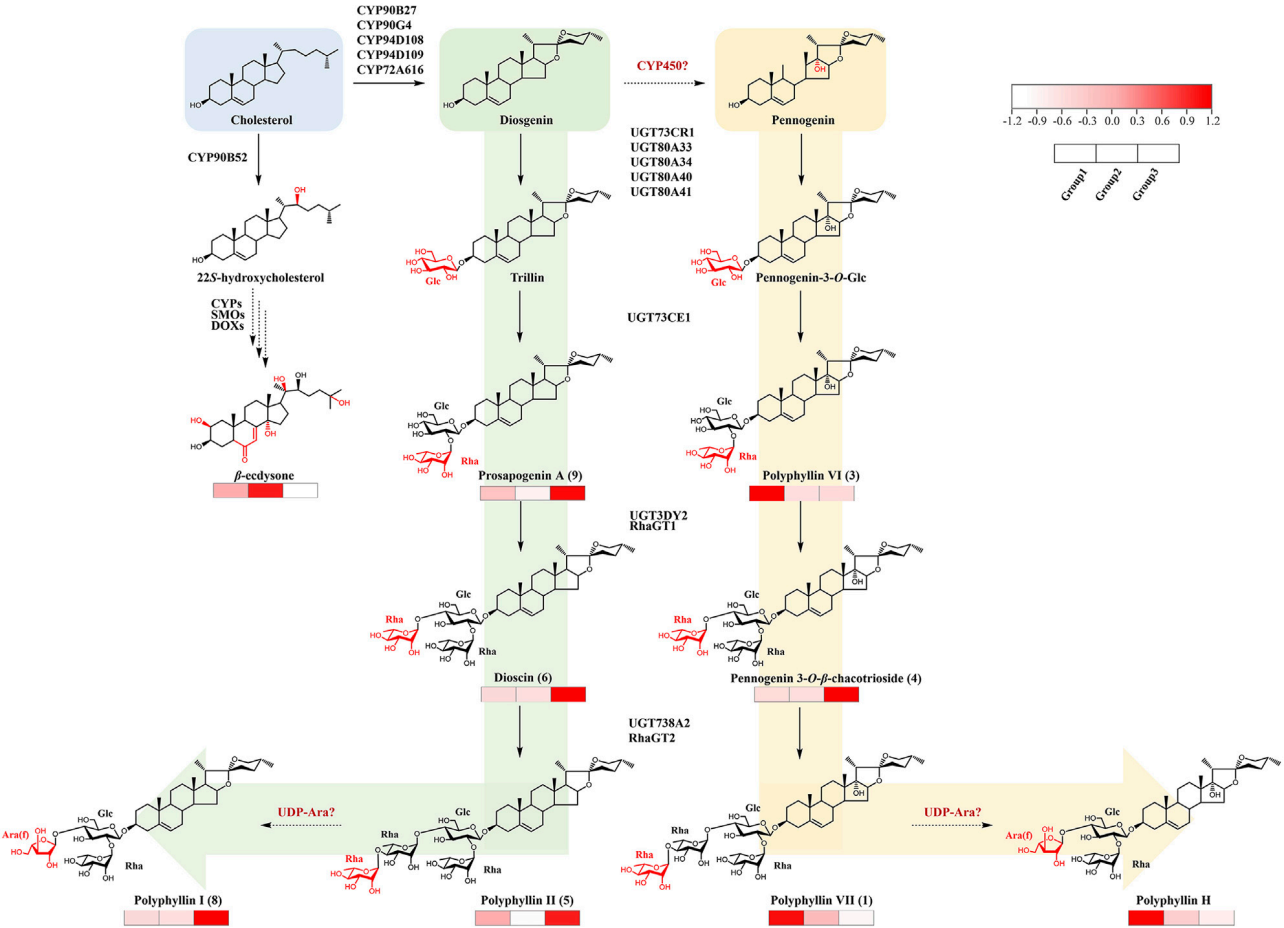
Proposed biosynthesis pathway of major steroidal compounds in *Paris* species. Solid arrows represent elucidated biosynthetic steps, while dashed arrows indicate hypothetical and unresolved steps. The blue background area marks the precursor Cholesterol. Green and yellow backgrounds denote the biosynthetic branches leading to diosgenin-type and pennogenin-type saponins, respectively. Heatmaps below each compound reflect the average relative abundance across three groups (Group1, Group2, and Group3) according to the color scale shown.

Synthetic biology through heterologous expression systems presents a promising strategy for efficient and sustainable production of steroidal saponins from *Paris* plants. Cholesterol, synthesized via the cytosolic mevalonate (MVA) pathway, serves as a common precursor for steroidal compounds (Yin et al., 2023). Cholesterol undergoes oxidative modifications catalyzed by cytochrome P450 enzymes (CYPs) to form two main aglycones, diosgenin and pennogenin. These aglycones subsequently glycosylated by uridine diphosphate glycosyltransferases (UGTs), producing a variety of steroidal saponins. To date, several CYPs and UGTs involved in Polyphyllin biosynthesis have been identified (Figure 6) (Zhou et al., 2024; Song et al., 2025; Zhao et al., 2025). However, some key steps remain unclear. In particular, the enzyme introducing the hydroxyl group is introduced at pennogenin’s C-17 position is still unknown. It remains to be determined whether this occurs via a CYP450 enzyme acting on diosgenin or earlier in the pathway before sterol cyclization (Hua et al., 2025). Additionally, the arabinofuranosyltransferase responsible for glycosylation at polyphyllin I’s C-4 position has yet to be identified.

Long-term evolutionary divergence and ecological adaptation have contributed to the diversification of metabolic networks among *Paris* species, resulting in distinct metabolomic profiles (Miao et al., 2024). In this study, a comprehensive analysis of 26 *Paris* species and varieties revealed significant interspecific differences in both the composition and relative abundance of metabolites. For example, species in Group 1 mainly accumulated pennogenin saponins, while Group 3 species exhibited higher levels of diosgenin saponins. These metabolic variations are likely to reflect variations in the activity or regulation of key biosynthetic enzymes or the redistribution of metabolic flux across competing pathways. Further investigation is needed to determine whether such differences stem from enzyme specificity, gene expression levels, or pathway branching. Integrating metabolomic with transcriptomic data may offer valuable insights into these questions, particularly the enzymatic basis of C-17 hydroxylation in pennogenin biosynthesis, and ultimately shed light on how metabolic evolution has shaped chemodiversity within the genus.

Despite the observed metabolic diversity, most existing studies on polyphyllin biosynthesis have focused on PPY, leaving other species largely unexplored. It is conceivable that alternative biosynthetic routes exist in other members of the genus, highlighting the need for future cross-species comparative studies to reveal alternative biosynthetic strategies. Notably, PX was found to accumulate significantly higher total levels of polyphyllin I, II, and VII compared to PPY and PPC, indicating its potential as an alternative model for investigating saponin biosynthesis. In addition, *β*-ecdysone was consistently detected across all seven official medicinal and newly identified *Paris* species in this study. As a plant steroid hormone analog, *β*-ecdysone plays a crucial role in regulating development and sterol metabolism (Wang Y. Z. et al., 2017). Although CYP90B52 has been identified as catalyzing the formation of 22S-hydroxycholesterol in the polyphyllin biosynthetic pathway (Yin et al., 2023), the enzymes responsible for other key hydroxylation steps, including those at the C-2, C-14, and C-25 positions remain unidentified.

## 5 Conclusion

This study employed UHPLC-Q-TOF/MS-based metabolomics to classify 26 *Paris* species and varieties into three distinct groups. The analysis revealed significant variations in the composition and concentration of two key bioactive compound types, pennogenin and diosgenin saponins, which may serve as chemical markers for metabolic differentiation and phylogenetic relationships within the genus. By combining pharmacophylogeny, the metabolomes of newly identified species were compared with those of pharmacopoeial species. This analysis identified *P. xuefengshanensis* and *P. tengchongensis* as promising candidates for antitumor drug development, while *P. qiliangiana* may exhibit hemostatic properties. These findings enhance the understanding of metabolic patterns, inform taxonomic classification, and support the medicinal evaluation of under-researched *Paris* species.

## Data Availability

The original contributions presented in the study are included in the article/Supplementary Material, further inquiries can be directed to the corresponding authors.
