# Supplementary material for: UHPLC-Q-TOF/MS unveils metabolic diversity in Paris L. and highlights medicinal potential of five newly identified species
Source: Front Pharmacol. 2025 Jul 23;16:1605264. doi: 10.3389/fphar.2025.1605264 (PMC12325426; doi:10.3389/fphar.2025.1605264)
Supplement: Supplementary file 1 [file DataSheet1.docx]

Supplementary Material

# Supplementary Tables

**Supplementary Table 1.** Sample information of *Paris* L. (n ≥ 3).

| **NO.** | **Species** | | | **Growth time (years)** | **Location**  (County/City, Province) | **Specimen code** | **Group in this study** |
| --- | --- | --- | --- | --- | --- | --- | --- |
|  | **Li Heng’s classification system** | **Generic name** | **Plants of the World Online** |  |  |  |  |
| 1 | *P. cronquistii* (Takht.) H. Li | Lingyun Chonglou | *P. cronquistii* (Takht.) H. Li | – | Xinning, Hunan | PCr-1 | Group 2-2 |
| 2 |  |  |  |  |  | PCr-2 |  |
| 3 |  |  |  |  |  | PCr-3 |  |
| 4 | *P. daliensis* H. Li et V. G. Soukup | Dali Chonglou | *P. yunnanensis* Franch. | 7-8 | Dali, Yunnan | PDa-1 | Group 2-1 |
| 5 |  |  |  | 9-10 | Dali, Yunnan | PDa-2 |  |
| 6 |  |  |  |  |  | PDa-3 |  |
| 7 | *P. delavayi* var. *delavayi* Franch. | Jinxian Chonglou | *P. delavayi* Franch. | 4 | Enshi, Hubei | PDe-1 | Group 2-2 |
| 8 |  |  |  | 6 |  | PDe-2 |  |
| 9 |  |  |  | 4-5 | Changsha, Hunan | PDe-3 |  |
| 10 | *P. dunniana* Lévl. | Hainan Chonglou | *P. dunniana* H. Lév. | 7 | Fangchenggang, Guangxi | PDu-1 | Group 2-2 |
| 11 |  |  |  | 3 |  | PDu-2 |  |
| 12 |  |  |  | 3-4 |  | PDu-3 |  |
| 13 |  |  |  | 7 | Wuzhishan, Hainan | PDu-4 |  |
| 14 |  |  |  | 7 |  | PDu-5 |  |
| 15 |  |  |  | 7 |  | PDu-6 |  |
| 16 | *P. fargesii* var. *brevipetalata* T.C.Huang & K.C.Yang | Duanban Qiuyaoge Chonglou | *P. fargesii* Franch. | 10 | Enshi, Hubei | PFB-1 | Group 1 |
| 17 |  |  |  |  |  | PFB-2 |  |
| 18 |  |  |  |  |  | PFB-3 |  |
| 19 | *P. fargesii* var. *fargesii* Franch. | Qiuyaoge Chonglou | *P. fargesii* Franch. | 8 | Enshi, Hubei | PF-1 | Group 1 |
| 20 |  |  |  |  |  | PF-2 |  |
| 21 |  |  |  |  |  | PF-3 |  |
| 22 | *P. fargesii* var. *latipetala* H.Li & V.G.Soukup | Kuanban Qiuyaoge Chonglou | *P. fargesii* Franch. | 5+ | Sangzhi, Hunan | PFL-1 | Group 1 |
| 23 |  |  |  | 8 | Xinning, Hunan | PFL-2 |  |
| 24 |  |  |  | 8 | Cili, Hunan | PFL-3 |  |
| 25 | *Paris fargesii* var. *petiolate* (Baker ex C. H. Wright) F. T. Wang & Tang | Jvbing Chonglou | *P. delavayi* Franch. | 15 | Shiyan, Hubei | PFP-1 | Group 2-1 |
| 26 |  |  |  |  |  | PFP-2 |  |
| 27 |  |  |  |  |  | PFP-3 |  |
| 28 | *P. forrestii* (Takht.) H. Li | Changzhu Chonglou | *P. forrestii* (Takht.) H. Li | 7-8 | Yunlong, Yunnan | PFo-1 | Group 2-1 |
| 29 |  |  |  | 5 |  | PFo-2 |  |
| 30 |  |  |  | 4 |  | PFo-3 |  |
| 31 | *P. mairei* H. Lév. | Mao Chonglou | *P. mairei* H. Lév. | – | Xinning, Hunan | PMai-1 | Group 2-2 |
| 32 |  |  |  | 3+ | Enshi, Hubei | PMai-2 |  |
| 33 |  |  |  | 3+ | Shiyan, Hubei | PMai-3 |  |
| 34 | *P. marmorata* Stearn | Huaye Chonglou | *P. marmorata* Stearn | – | Guiyang, Hunan | PMar-1 | Group 2-2 |
| 35 |  |  |  |  |  | PMar-2 |  |
| 36 |  |  |  |  |  | PMar-3 |  |
| 37 | *P. polyphylla* var. *appendiculata* Hara | Duangeng Chonglo*u* | *P. thibetica* Franch. | 6+ | Enshi, Hubei | PPA-1 | Group 1 |
| 38 |  |  |  |  |  | PPA-2 |  |
| 39 |  |  |  | 8-9 | Enshi, Hubei | PPA-3 |  |
| 40 | *P. polyphylla* var. *chinensis* (Franch.) Hara | Qiye Yizhihua | *P. chinensis* Franch. | 6+ | Xinning, Hunan | PPC-1 | Group 2-1 |
| 41 |  |  |  | 8+ |  | PPC-2 |  |
| 42 |  |  |  | 9 | Xinhua, Hunan | PPC-3 |  |
| 43 | *P. polyphylla* var. *nana* H. Li | Ai Chonglou | *P. yunnanensis* Franch. | 13 | Enshi, Hubei | PPN-1 | Group 2-2 |
| 44 |  |  |  | 9 |  | PPN-2 |  |
| 45 |  |  |  | 6 |  | PPN-3 |  |
| 46 | *P. polyphylla* var. *polyphylla* Smith | Duoye Chonglou | *P. polyphylla* Smith | – | Xinning, Hunan | PP-1 | Group 2-2 |
| 47 |  |  |  |  |  | PP-2 |  |
| 48 |  |  |  | 9+ | Enshi, Hubei | PP-3 |  |
| 49 | *P. polyphylla* var. *pseudothibetica* H. Li | Changyaoge Chonglou | *P. delavayi* Franch. | 12+ | Hongjiang, Hunan | PPP-1 | Group 3-2 |
| 50 |  |  |  | – |  | PPP-2 |  |
| 51 |  |  |  | 8-10 |  | PPP-3 |  |
| 52 | *P. polyphylla* var. *stenophylla* Franch. | Xiaye Chonglou | *P. lancifolia* Hayata | – | Xinning, Hunan | PPS-1 | Group 2-2 |
| 53 |  |  |  | 6 | Guidong, Hunan | PPS-2 |  |
| 54 |  |  |  |  |  | PPS-3 |  |
| 55 | *P. polyphylla* var. *yunnanensis* (Ftanch.) Hand. -Mazz | Dian Chonglou | *P. yunnanensis* Franch. | 9 | Guiyang, Hunan | PPY-1 | Group 3-1 |
| 56 |  |  |  | 6+ |  | PPY-2 |  |
| 57 |  |  |  | – |  | PPY-3 |  |
| 58 | *P. thibetica* Franch. | Heizi Chonglou | *P. thibetica* Franch. | 6 | Cili, Hunan | PTh-1 | Group 2-2 |
| 59 |  |  |  | 8 | Enshi, Hubei | PTh-2 |  |
| 60 |  |  |  |  |  | PTh-3 |  |
| 61 | *P. verticillate* M. Bieb. | Bei Chonglou | *P. verticillate* M. Bieb. | 5 | Changbai Mountains, Jilin | PVe-1 | Group 2-2 |
| 62 |  |  |  | 6 |  | PVe-2 |  |
| 63 |  |  |  | 6 |  | PVe-3 |  |
| 64 | *P. vietnamensis* (Takht.) H. Li | Nan Chonglou | *P. vietnamensis* (Takht.) H. Li | 3 | Honghe, Yunnan | PVi-1 | Group 3-2 |
| 65 |  |  |  | 3 |  | PVi-2 |  |
| 66 |  |  |  | 3 |  | PVi-3 |  |
| **Newly Identified Species** | | | | | | | |
| 67 | *P. nitida* G.W. Hu, Z.Wang & Q.F.Wang | Liangye Chonglou | *P. caobangensis* Y.H.Ji, H.Li & Z.K.Zhou | – | Xinning, Hunan | PN-1 | Group 2-2 |
| 68 |  |  |  | 4 | Liuyang, Hunan | PN-2 |  |
| 69 |  |  |  | 7 |  | PN-3 |  |
| 70 | *P. qiliangiana* H. Li, J. Yang et Y. H. Wang | Qiliang Chonglou | *P. qiliangiana* H. Li, J. Yang et Y. H. Wang | 7-9 | Zhangjiajie, Hunan | PQ-1 | Group 1 |
| 71 |  |  |  | 4+ | Enshi, Hubei | PQ-2 |  |
| 72 |  |  |  |  |  | PQ-3 |  |
| 73 | *P. tengchongensis* Y. H. Ji, C. J. Yang & Yu L. Huang | Tengchong Chonglou | *P. forrestii* (Takht.) H. Li | 7 | Wuzhishan, Hainan | PTe-1 | Group 3-1 |
| 74 |  |  |  |  |  | PTe-2 |  |
| 75 |  |  |  |  |  | PTe-3 |  |
| 76 | *P. xuefengshanensis* Z.Wang, H.Li & G.W.Hu | Xuefengshan Chonglou | – | 5-6 | Xinning, Hunan | PX-1 | Group 3-2 |
| 77 |  |  |  |  |  | PX-2 |  |
| 78 |  |  |  | – | Xinning, Hunan | PX-3 |  |
| 79 | *P. yanchii* H.Li, L.G.Lei & Y.M.Yang | Yunlong Chonglou | *P. yanchii* H.Li, L.G.Lei & Y.M.Yang | 5-6 | Dali, Yunnan | PYa-1 | Group 2-2 |
| 80 |  |  |  |  |  | PYa-2 |  |
| 81 |  |  |  |  |  | PYa-3 |  |
| **Outgroups** | | | | | | |  |
| 82 | *Trillium tschonoskii* Maxim. |  | *Trillium tschonoskii* Maxim. | 5-6 | Enshi, Hubei | TT-1 | – |
| 83 |  |  |  |  |  | TT-2 |  |
| 84 |  |  |  |  |  | TT-3 |  |

**Note:**

- In order to fully explore the metabolic diversity of the subspecies samples, Li Heng’s classification system was used in this study and compared with the WFO classification system to ensure the correctness of sample sources.
- 5 new taxonomic species (published 2017-2024) not in Li Heng's classification system (1998); In the latest WFO, *P. xuefengshanensis* (2024, NO. 76) has not yet been received, *P. nitida* (NO. 67) and *P. tengchongensis* (NO. 73) were merged into *P. caobangensis* Y.H.Ji, H.Li & Z.K.Zhou and *P. forrestii* (Takht.) H. Li, respectively. The present study retained the original classification and name in order to explore the metabolic diversity and resource utilization of the genus.
- The growth time (years) of samples was estimated based on the number of stem traces on rhizomes. In a few samples, the traces were incomplete or unclear, which hindered the prediction of growth time.
- Li Heng's classification system sees Li, H. (1998). The genus *Paris* (Trilliaceae). Beijing: Science Press.
- Plants of the World Online see <https://powo.science.kew.org/taxon/urn:lsid:ipni.org:names:24614-1>

**Supplementary Table 2.** Identification results of the 10 reference standards.

| **Peak** | **RT (min)** | **[M-H]^-^measured** | **Adducts** | **Compound name** | **Formula** | **Molecular weight** | **Mass error(ppm)** | **Type** |
| --- | --- | --- | --- | --- | --- | --- | --- | --- |
| 1 | 37.57 | 1029.5281 | 1065.5067[M+Cl]^-^ 1075.5340[M+HCOO]^-^ 1123.4629[M+93]^-^ 2097.0367[2M+Cl]^-^ | Polyphyllin VII | C₅₁H₈₂O₂₁ | 1030.5349 | 0.4857 | Pennogenin |
| 2 | 37.91 | 899.4643 | 935.4432[M+Cl]^-^ 945.4714[M+HCOO]^-^ 993.3993[M+93]^-^ 1836.9165[2M+Cl]^-^ 1065.5045[M+165]^-^ | 17-Hydroxygracillin | C₄₅H₇₂O₁₈ | 900.4719 | -0.3335 | Pennogenin |
| 3 | 38.90 | 737.4112 | 773.3898[M+Cl]^-^ 783.4187[M+HCOO]^-^ 1511.8086[2M+Cl]^-^ 835.3782[M+97]^-^ | Polyphyllin VI | C₃₉H₆₂O₁₃ | 738.4190 | -0.8137 | Pennogenin |
| 4 | 39.98 | 919.4467 | 919.4477[M+Cl]^-^ 929.4758[M+HCOO]^-^ 1804.9265[2M+Cl]^-^ 977.4050[M+93]^-^ | Pennogenin 3-O-*β*-chacotrioside | C₄₅H₇₂O₁₇ | 884.4770 | 0.3263 | Pennogenin |
| 5 | 43.09 | 1013.5335 | 1049.5120[M+Cl]^-^ 1059.5396[M+HCOO]^-^ 2065.0486[2M+Cl]^-^ 1107.4681[M+93] | Polyphyllin II | C₅₁H₈₂O₂₀ | 1014.5399 | 0.7893 | Dioscin |
| 6 | 43.66 | 867.4742 | 903.4530[M+Cl]^-^ 913.4813[M+HCOO]^-^ 961.4099[M+93]^-^ 1035.4939[M+167]^-^ 1771.9340[2M+Cl]- | Dioscin | C₄₅H₇₂O₁₆ | 868.4820 | -0.6917 | Dioscin |
| 7 | 43.93 | 883.4697 | 919.4483[M+Cl]^-^ 929.4764[M+HCOO]^-^ 1804.9267[2M+Cl]^-^ 977.4048[M+93]^-^ | Gracillin | C₄₅H₇₂O₁₇ | 884.4770 | 0.0000 | Dioscin |
|  |  |  |  | Diosgenin-3-O-Rha(1→2)-[Glc(1→6)]-Glc |  |  |  |  |
| 8 | 44.21 | 853.4593 | 889.4374[M+Cl]^-^ 899.4658[M+HCOO]^-^ 1744.9074[2M+Cl]^-^ | Polyphyllin I | C₄₄H₇₀O₁₆ | 854.4664 | 0.2343 | Dioscin |
| 9 | 45.06 | 721.4167 | 757.3947[M+Cl]^-^ 767.4232[M+HCOO]^-^ 1479.8190[2M+Cl]^-^ | Prosapogenin A | C₃₉H₆₂O₁₂ | 722.4241 | -0.2772 | Dioscin |

Note: All mass errors were calculated using the [M−H]⁻ ion, except for Pennogenin 3-O-*β*-chacotrioside, where the [M−H]⁻ ion was not detected for peak 4, the [M+Cl]⁻ ion was used instead.

**Supplementary Table 3.** Identification and relative quantitative analysis of 24 common metabolites among seven *Paris* species.

| **No.** | **RT (min)** | **Common Metabolites** | **Type** | **Distribution and Peak Area (×10^8^)** | | | | | | |
| --- | --- | --- | --- | --- | --- | --- | --- | --- | --- | --- |
|  |  |  |  | **PPC** | **PPY** | **PN** | **PQ** | **PX** | **PYa** | **PTe** |
| 1 | 9.61 | *β*-ecdysone or its isomer | insect allergy hormones | 0.4608 | 1.1173 | 0.6869 | 0.2323 | 0.0707 | 0.0533 | 0.4733 |
| 2 | 12.65 | Paripolin G or Paripolin H | spirostanol saponins | 0.0443 | 0.0606 |  | - | - | - | - |
| 3 | 19.12 | Parisverticoside A or Parispolyoside C | spirostanol saponins | 0.1176 | - | - | - | - | - | 0.6342 |
| 4 | 19.32 | Chonglouoside SL-2 | furostanol saponins | 0.5774 | - | - | 0.2552 | - | - | 0.1432 |
| 5 | 19.76 | Parisyunnanoside A | furostanol saponins | 2.5622 | 0.7779 | - | 1.3318 | - | 0.2249 | 0.8430 |
| 6 | 19.95 | Saponin Th or its isomer | furostanol saponins | 1.5903 | 1.0085 | 0.5098 | 0.5165 | - | 0.4922 | 1.0869 |
| 7 | 2.23 | Parpetioside C | furostanol saponins | 0.9524 | - | - | 0.9428 | - | - | 2.4527 |
| 8 | 25.23 | Dichotomin | furostanol saponins | 0.0618 | 2.0365 | 1.6786 | - | 0.9799 | - | 0.5583 |
| 9 | 25.43 | Parisaponin I or Polyphyllin G | furostanol saponins | 3.5196 | 0.2186 | - | - | 1.1046 | - | 0.4244 |
| 10 | 25.81 | Protogracillin | furostanol saponins | 0.0831 | - | - | - | 1.8063 | - | 6.5161 |
| 11 | 30.00 | Parisvanioside C or its isomer | isospirostanol saponins | 0.0950 | - | - | - | 0.1318 | - | - |
| 12 | 36.56 | Ypsiyunnoside D | cholestanol saponins | 0.1626 | - | - | - | - | 0.0208 | - |
| 13 | 36.86 | Parispolyoside A or Parisvanioside B | cholestanol saponins | 0.4045 | 0.1277 | - | - | - | - | - |
| 14 | 37.28 | Kingianoside K | isospirostanol saponins | 0.5691 | - | - | 1.3984 | - | - | - |
| 15 | 37.67 | Polyphyllin VII | isospirostanol saponins (pennogenin) | 2.4895 | 1.2827 | 0.5406 | 1.4897 | 0.1813 | 0.1035 | 0.8244 |
| 16 | 37.93 | 17-Hydroxygracillin | isospirostanol saponins (pennogenin) | 2.1966 | - | - | 1.9330 | - | 0.0202 | 2.2296 |
| 17 | 38.39 | Polyphyllin H or its isomer | isospirostanol saponins (pennogenin) | 3.8188 | 0.7827 | - | 2.8346 | 0.0573 | 0.0561 | 0.7550 |
| 18 | 38.93 | Polyphyllin VI | isospirostanol saponins (pennogenin) | 0.4594 | - | - | 3.1614 | - | - | - |
| 19 | 40.28 | Saponin Tc or its isomer | isospirostanol saponins (pennogenin) | 0.0362 | - | 0.0333 | - | - | - | - |
| 20 | 43.90 | Polyphyllin II | isospirostanol saponins (diosgenin) | 0.1781 | 1.6749 | 0.8045 | - | 2.9841 | - | 0.5316 |
| 21 | 43.66 | Dioscin | isospirostanol saponins (diosgenin) | 0.0656 | - | - | 0.1312 | 1.1630 | - | - |
| 22 | 43.94 | Gracillin | isospirostanol saponins (diosgenin) | 0.2956 | 0.6714 | - | 0.6125 | 3.4823 | - | 5.4712 |
| 23 | 44.19 | Polyphyllin I | isospirostanol saponins (diosgenin) | 0.5794 | 1.9487 | - | 0.3199 | 1.7555 | - | 0.2697 |
| 24 | 45.00 | Prosapogenin A | isospirostanol saponins (diosgenin) | - | 0.2929 | - | 0.6158 | 3.9374 | - | 0.1847 |
| **Total Polyphyllin I, II, VII** | | | | 3.2470 | 4.9063 | 1.3451 | 1.8096 | 4.9209 | 0.1035 | 1.6257 |

**Abbreviations:** PPC, *P. polyphylla* var. *chinensis*; PPY, *P. polyphylla* var. *yunnanensis*; PN, *P. nitida*; PQ, *P. qiliangiana*; PX, *P. xuefengshanensis*; PYa, *P. yanchii*; PTe, *P. tengchongensis*.

**Supplementary Table 4.** Identification analysis of 43 DAMs among seven *Paris* species.

| **NO.** | **rt(min)** | **DAMs** | **Formula** | **[M]^-^measured** | **mass error(ppm)** | **Type** | **Variable Importance in Projection** | | | | | | | | | | |
| --- | --- | --- | --- | --- | --- | --- | --- | --- | --- | --- | --- | --- | --- | --- | --- | --- | --- |
|  |  |  |  |  |  |  | **PPC_vs_PPY** | **PPC_vs_PN** | **PPC_vs_PQ** | **PPC_vs_PX** | **PPC_vs_PYa** | **PPC_vs_PTe** | **PPY_vs_PN** | **PPY_vs_PQ** | **PPY_vs_PX** | **PPY_vs_PYa** | **PPY_vs_PTe** |
| P1 | 9.03 | Parisyunnanoside G or its ismoer | C₅₆H₈₈O₂₉ | 1223.5340 | 0.0817 | spirostanol saponins | - | - | - | - | 2.21 | - | - | - | - | - | 1.89 |
| P2 | 10.77 | Parisyunnanoside K or Parisyunnanoside L | C₅₅H₈₆O₂₈ | 1193.5253 | 1.6757 | spirostanol saponins | - | - | - | 1.88 | 2.47 | 2.14 | - | - | - | 1.85 | - |
| P3 | 11.14 | Parisyunnanoside H or its isomer | C₅₀H₇₈O₂₄ | 1061.4821 | 1.0363 | spirostanol saponins | - | - | - | - | 2.69 | - | - | - | - | 2.47 | - |
| P4 | 13.09 | Parispolyoside D | C₄₅H₇₂O₂₀ | 967.4328 | 1.7572 | spirostanol saponins | - | - | - | - | - | - | - | - | - | - | 1.92 |
| P5 | 14.29 | Padelaoside B | C₅₀H₈₀O₂₄ | 1063.4980 | 1.2224 | spirostanol saponins | 1.80 | - | - | - | 1.85 | - | - | - | - | - | - |
| P6 | 15.05 | Chonglouoside SL-18 | C₄₅H₇₂O₂₀ | 967.4332 | 2.1707 | spirostanol saponins | - | - | - | - | - | - | - | - | - | - | 2.04 |
| P7 | 17.60 | parisfargoside SL-1 or Parisfargoside SL-4 | C₅₀H₈₀O₂₃ | 1047.5030 | 1.1456 | spirostanol saponins | - | 1.88 | - | - | - | - | - | - | - | - | - |
| P8 | 18.83 | Parpetioside B | C₅₅H₉₀O₂₇ | 1181.5626 | 2.4544 | furostanol saponins | - | - | 2.24 | - | - | - | - | - | - | - | - |
| P9 | 19.12 | Parisverticoside A or Parispolyoside C | C₄₄H₇₀O₁₉ | 901.4452 | 1.4421 | spirostanol saponins | 1.91 | - | 1.91 | - | - | - | - | - | - | - | 2.28 |
| P10 | 19.16 | Chonglouoside SL-20 | C₅₁H₈₄O₂₃ | 1063.5326 | -0.4701 | furostanol saponins | 2.12 | - | - | - | - | - | - | - | - | - | - |
| P11 | 19.58 | Parisyunnanoside A | C₅₀H₈₂O₂₃ | 1049.5217 | 4.0971 | furostanol saponins | - | 1.80 | - | - | 1.82 | - | 1.89 | - | 1.81 | - | - |
| P12 | 20.08 | Parpetioside C | C₅₁H₈₄O₂₄ | 1079.5314 | 3.1495 | furostanol saponins | - | - | 1.85 | - | - | - | - | - | - | - | 2.45 |
| P13 | 20.09 | Saponin Th or its isomer | C₅₇H₉₄O₂₇ | 1209.5931 | 1.7361 | furostanol saponins | 1.84 | - | 1.85 | - | - | - | - | - | 1.87 | - | 2.41 |
| P14 | 24.35 | Pennogenin-3-O-Glc(1→5)-Ara(1→4)-[Rha(1→2)]-Glc | C₅₀H₈₀O₂₂ | 1031.5086 | 1.7450 | isospirostanol saponins (pennogenin) | - | - | - | - | - | - | 1.83 | - | - | - | - |
| P15 | 24.78 | Chonglouoside SL-3 or its isomer | C₅₁H₈₂O₂₃ | 1061.5199 | 2.3551 | spirostanol saponins | - | - | - | - | - | 1.83 | - | - | - | - | - |
| P16 | 24.96 | Dichotomin | C₅₇H₉₄O₂₆ | 1193.6001 | 3.3512 | furostanol saponins | - | - | - | - | - | 2.00 | - | 1.88 | - | 2.00 | - |
| P17 | 25.05 | Protodioscin | C₅₁H₈₄O₂₂ | 1047.5396 | 1.4319 | furostanol saponins | 2.13 | - | - | - | - | - | - | - | - | - | - |
| P18 | 25.23 | Parisaponin I or Polyphyllin G | C₅₀H₈₂O₂₂ | 1033.5254 | 2.8059 | furostanol saponins | 1.89 | - | - | - | - | - | 2.22 | - | - | 2.12 | 1.95 |
| P19 | 25.73 | Protogracillin | C₅₁H₈₄O₂₃ | 1063.5371 | 3.7610 | furostanol saponins | - | - | - | - | - | - | 2.03 | - | - | 2.11 | - |
| P20 | 25.79 | Protoprogenin II or Trigofoenoside A | C₄₅H₇₄O₁₈ | 901.4831 | 3.2169 | furostanol saponins | - | - | - | - | - | - | - | - | - | 2.05 | - |
| P21 | 27.48 | Parisfargoside B | C₄₄H₆₈O₁₇ | 867.4433 | 5.6488 | cholestanol saponins | - | - | - | - | - | 1.86 | - | - | - | - | - |
| P22 | 29.24 | Smilaxchinoside B or its isomer | C₅₇H₉₂O₂₆ | 1191.5822 | 1.5106 | furostanol saponins | 1.92 | - | - | - | - | - | - | - | - | - | 1.93 |
| P23 | 31.45 | Parisverticoside B | C₄₄H₇₀O₁₈ | 885.4505 | 1.8070 | isospirostanol saponins | - | - | - | - | - | 1.87 | - | - | - | - | - |
| P24 | 31.90 | Parisyunnanoside B | C₅₀H₈₀O₂₁ | 1015.5146 | 2.6588 | furostanol saponins | 1.80 | - | - | - | - | - | 2.16 | - | - | - | - |
| P25 | 32.56 | Parisfargoside SL-7 or its isomer | C₅₁H₈₀O₂₂ | 1043.5086 | 1.7250 | spirostanol saponins | - | - | - | - | - | - | - | - | - | - | 1.86 |
| P26 | 34.28 | Pariposide A or its isomer | C₃₉H₆₀O₁₄ | 787.3695 | 2.2861 | isospirostanol saponins | - | - | 1.86 | - | - | - | - | - | - | - | - |
| P27 | 36.67 | Polyphylloside F or its isomer | C₄₅H₇₂O₁₉ | 915.4572 | -2.5124 | isospirostanol saponins | - | - | - | - | - | - | - | - | - | - | 1.81 |
| P28 | 36.85 | Parispolyoside A or Parisvanioside B | C₄₅H₇₀O₁₈ | 897.4500 | 1.2257 | isospirostanol saponins | 1.80 | - | - | - | - | - | - | - | - | - | 1.80 |
| P29 | 37.26 | Kingianoside K | C₄₄H₆₈O₁₇ | 867.4395 | 1.2681 | isospirostanol saponins | 2.02 | 1.90 | - | - | - | 2.14 | - | - | - | - | - |
| P30 | 37.38 | Pennogenin-3-O-Rha(1→2)-[Xyl(1→5)-Aral(1→4)]-Glc | C₄₉H₇₈O₂₁ | 1001.4990 | 2.6960 | isospirostanol saponins (pennogenin) | - | - | 2.29 | - | - | - | - | 1.89 | - | - | - |
| P31 | 37.80 | Parisfargoside A or its isomer | C₃₉H₆₀O₁₃ | 735.3969 | 1.0878 | cholestanol saponins | - | - | 1.86 | - | - | - | - | 1.81 | - | - | - |
| P32 | 37.81 | Polyphyllin VI | C₃₉H₆₂O₁₃ | 737.4134 | 2.1698 | isospirostanol saponins (pennogenin) | - | - | 2.10 | - | - | - | - | 1.82 | - | - | - |
| P33 | 37.90 | 17-Hydroxygracillin | C₄₅H₇₂O₁₈ | 899.4660 | 1.5565 | isospirostanol saponins (pennogenin) | 2.09 | 1.89 | - | - | 1.81 | - | - | 1.85 | - | - | 2.55 |
| P34 | 40.44 | Parisvanioside D | C₄₀H₆₄O₁₄ | 803.4006 | 1.9915 | isospirostanol saponins | - | - | - | - | - | - | - | - | 1.86 | - | - |
| P35 | 42.65 | Polyphylloside III or its isomer | C₅₁H₈₂O₂₂ | 1045.524 | 1.4347 | spirostanol saponins | - | - | - | - | - | 2.10 | - | - | - | - | 1.88 |
| P36 | 43.05 | Polyphyllin II | C₅₁H₈₂O₂₀ | 1013.5363 | 3.5519 | isospirostanol saponins (diosgenin) | 2.14 | - | 2.17 | - | - | - | - | 2.50 | - | 2.20 | - |
| P37 | 43.43 | Dioscin | C₄₅H₇₂O₁₆ | 867.4766 | 2.0750 | isospirostanol saponins (diosgenin) | - | - | - | - | - | - | - | - | 1.87 | - | - |
| P38 | 43.46 | Reclinatoside | C₅₀H₈₀O₂₀ | 999.5192 | 2.2011 | isospirostanol saponins (diosgenin) | 2.06 | - | - | - | - | - | 2.00 | 1.96 | - | 1.99 | 1.81 |
| P39 | 43.87 | Gracillin | C₄₅H₇₂O₁₇ | 883.4696 | -0.1132 | isospirostanol saponins (diosgenin) | - | - | - | - | - | 1.95 | 1.91 | - | 1.95 | 1.88 | - |
| P40 | 44.15 | Polyphyllin I | C₄₄H₇₀O₁₆ | 853.4607 | 1.8747 | isospirostanol saponins (diosgenin) | 1.93 | - | - | - | - | - | 2.48 | 1.82 | - | 2.19 | - |
| P41 | 44.93 | Polyphyllin D or Diosgenin-3-O-Api(1→3)-[Rha(1→2)]-Glc | C₄₄H₇₀O₁₆ | 853.4609 | 2.1091 | isospirostanol saponins (diosgenin) | - | - | - | - | - | 1.95 | - | - | - | - | - |
| P42 | 44.99 | Prosapogenin A or its isomer | C₃₉H₆₂O₁₂ | 721.4181 | 1.6634 | isospirostanol saponins (diosgenin) | 2.01 | - | 1.82 | 2.02 | - | 2.05 | - | - | 1.99 | - | - |
| P43 | 45.23 | Sansevierin A or its isomer | C₃₉H₆₂O₁₃ | 737.4124 | 0.8137 | isospirostanol saponins | - | - | - | - | - | 2.02 | - | - | - | - | - |

**Abbreviations:** DAMs, differentially accumulated metabolites; PPC, *P. polyphylla* var. *chinensis*; PPY, *P. polyphylla* var. *yunnanensis*; PN, *P. nitida*; PQ, *P. qiliangiana*; PX, *P. xuefengshanensis*; PYa, *P. yanchii*; PTe, *P. tengchongensis*.

# Supplementary Figures


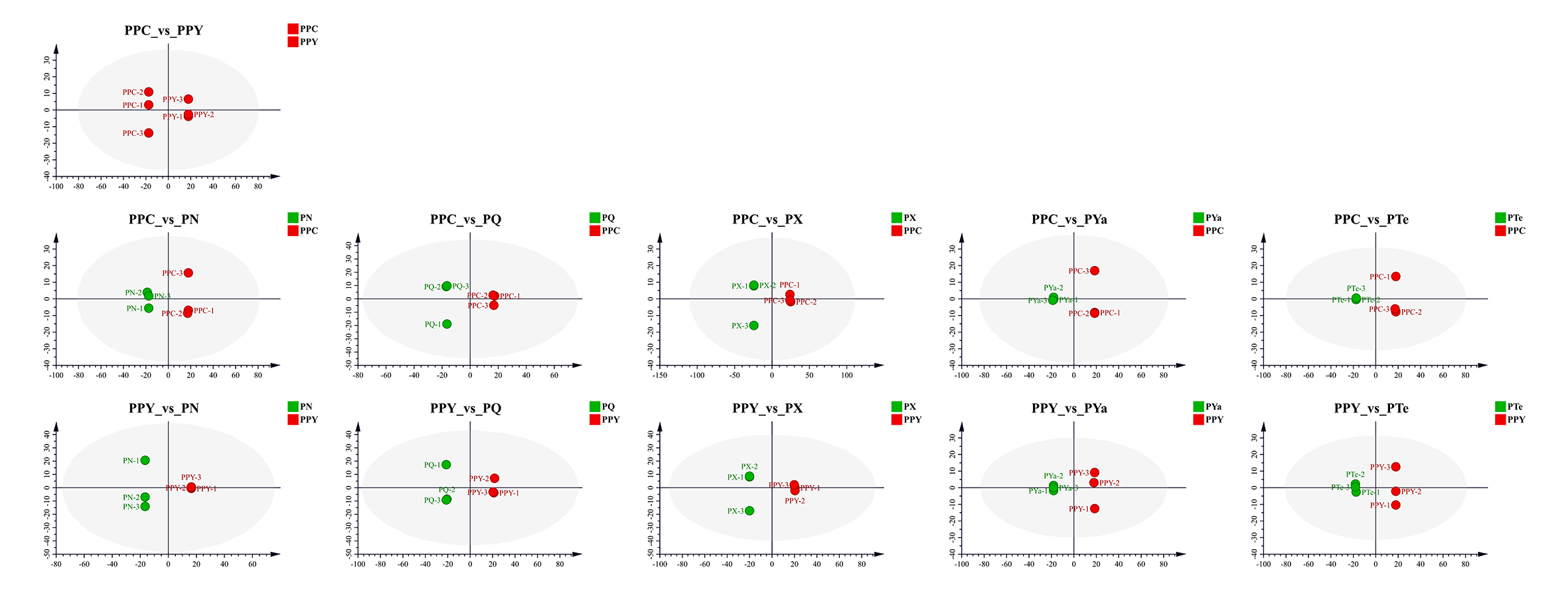


**Supplementary Figure 1.** OPLS-DA analysis of different *Paris* species

The OPLS-DA analysis results comparing *P. polyphylla var. chinensis* (PPC) and *P. polyphylla var. yunnanensis* (PPY) with *P. nitida* (PN), *P. qiliangiana* (PQ), *P. xuefengshanensis* (PX), *P. yanchii* (PYa), and *P. tengchongensis* (PTe) are displayed, illustrating the metabolic differences among the species. Each dot represents a sample, where red denotes PPC or PPY, and green represents PN, PQ, PX, PYa, or PTe. The ellipses represent Hotelling's T² (95%) confidence intervals, reflecting the distribution of samples within the predicted range of the model.
